# Speculations on the Evolution of Tail Regeneration in Lizards: Lessons from an Outstanding Case of Organ Regeneration in Amniotes

**DOI:** 10.3390/jdb14030035

**Published:** 2026-08-05

**Authors:** Lorenzo Alibardi

**Affiliations:** Comparative Histolab, 35127 Padova, Italy; loalibardi@gmail.com

**Keywords:** lizards, tail autotomy, amphibians, regeneration, evolution

## Abstract

Based on updated information on tail regeneration in lizards, a hypothesis is introduced to explain why these reptiles evolved their regenerative ability. The hypothesis, supported by paleontological evidence, considers tail regeneration associated with autotomy since the Paleozoic in amphibian anthracosaurs of the Carboniferous Period. It is submitted that these ancient amphibians developed tails containing stem cells localized in autotomous planes and/or inter-muscle connective tissues, from which they could regenerate their tails, as some extant salamanders do. The process of stem cell distribution during vertebrate development in salamanders and lizards, however, remains to be demonstrated. It is here suggested that autotomy and regeneration were inherited in the reptiliamorph basal amniotes of the Upper Carboniferous, then in captorhinids of the Permian, in eosuchians of the Triassic, and in the derived Mesozoic lizards, allowing survival and radiation into numerous families. Only in a few lizard families, perhaps under specific ecological adaptations, was autotomy lost, reducing or eliminating tail regeneration. Transcriptome analysis of developing versus regenerating tail indicates numerous differences, suggesting that regeneration utilizes alternative developmental gene pathways from those activated during development. Clarification of gene networks evolved for lizard tail regeneration may indicate the essential steps necessary to regenerate organs also in other amniotes.

## 1. Introduction to Tail Autotomy in Lizards

**Figure 1:**
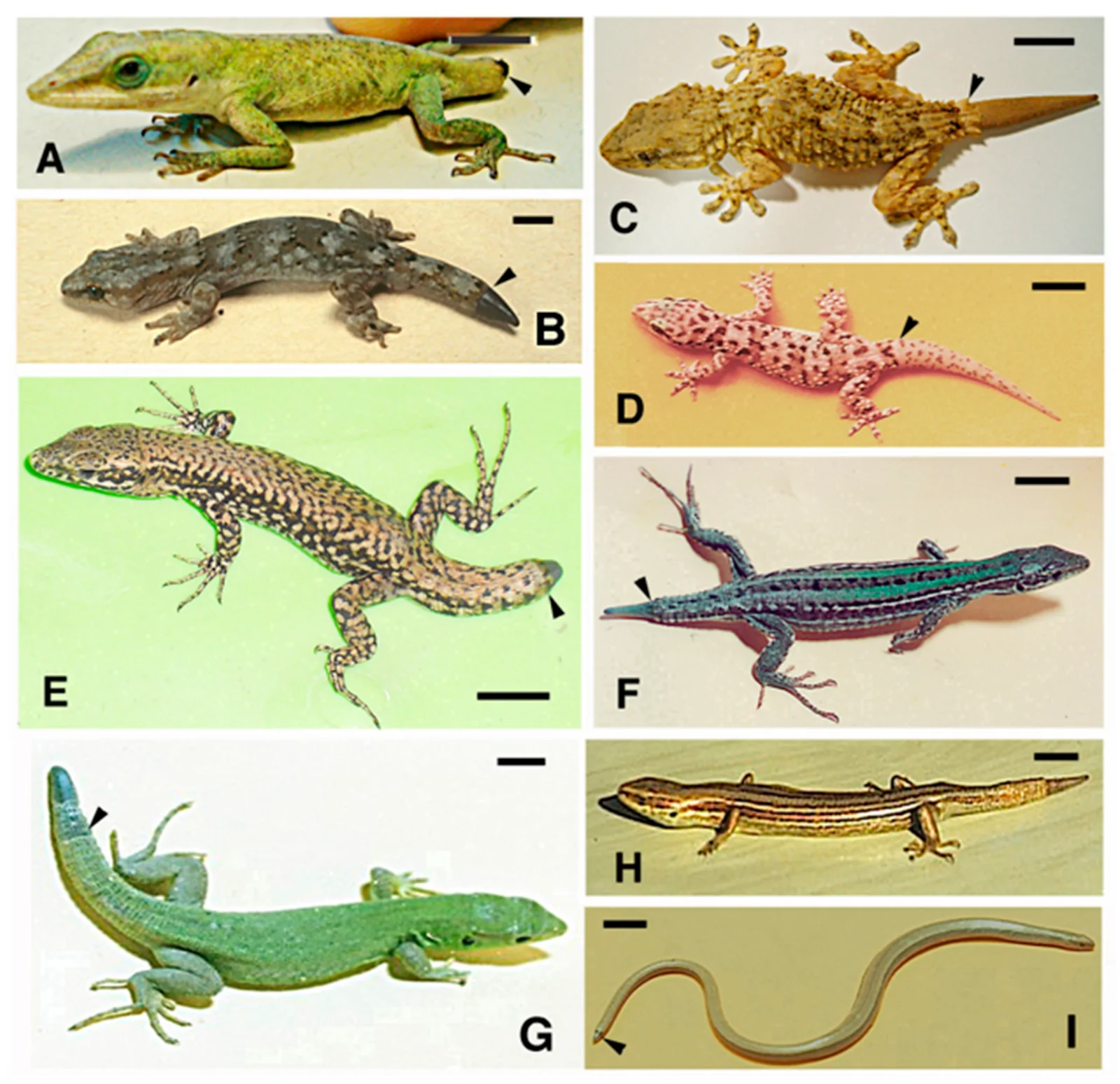
Some examples of tail regeneration in lizards. (**A**) *Anolis carolinensis* (iguanids) blastema (arrowhead); bar, 10 mm. (**B**) *Hoplodactylus maculatum* (gekkonids) cone (arrowhead); bar, 5 mm. (**C**) *Tarentula mauritanica* (gekkonids) largely regenerated tail (arrowhead); bar, 10 mm. (**D**) *Hemidactylus turcicus* (gekkonids) with regenerated tail (arrowhead); bar, 10 mm. (**E**) *Podarcis muralis* (lacertids) with blastema-cone (arrowhead); bar, 10 mm. (**F**), *Podarcis sicula* (lacertids) with elongating cone (arrowhead); bar, 10 mm. (**G**) *Lacerta viridis* (lacertids) with elongating tail (arrowhead); bar, 10 mm. (**H**) *Lampropholis delicata* (scincids) with elongating cone; bar, 5 mm. (**I**), *Anguis fragilis* (anguids) with regenerating cone (arrowhead); bar, 10 mm.

**Figure 2:**
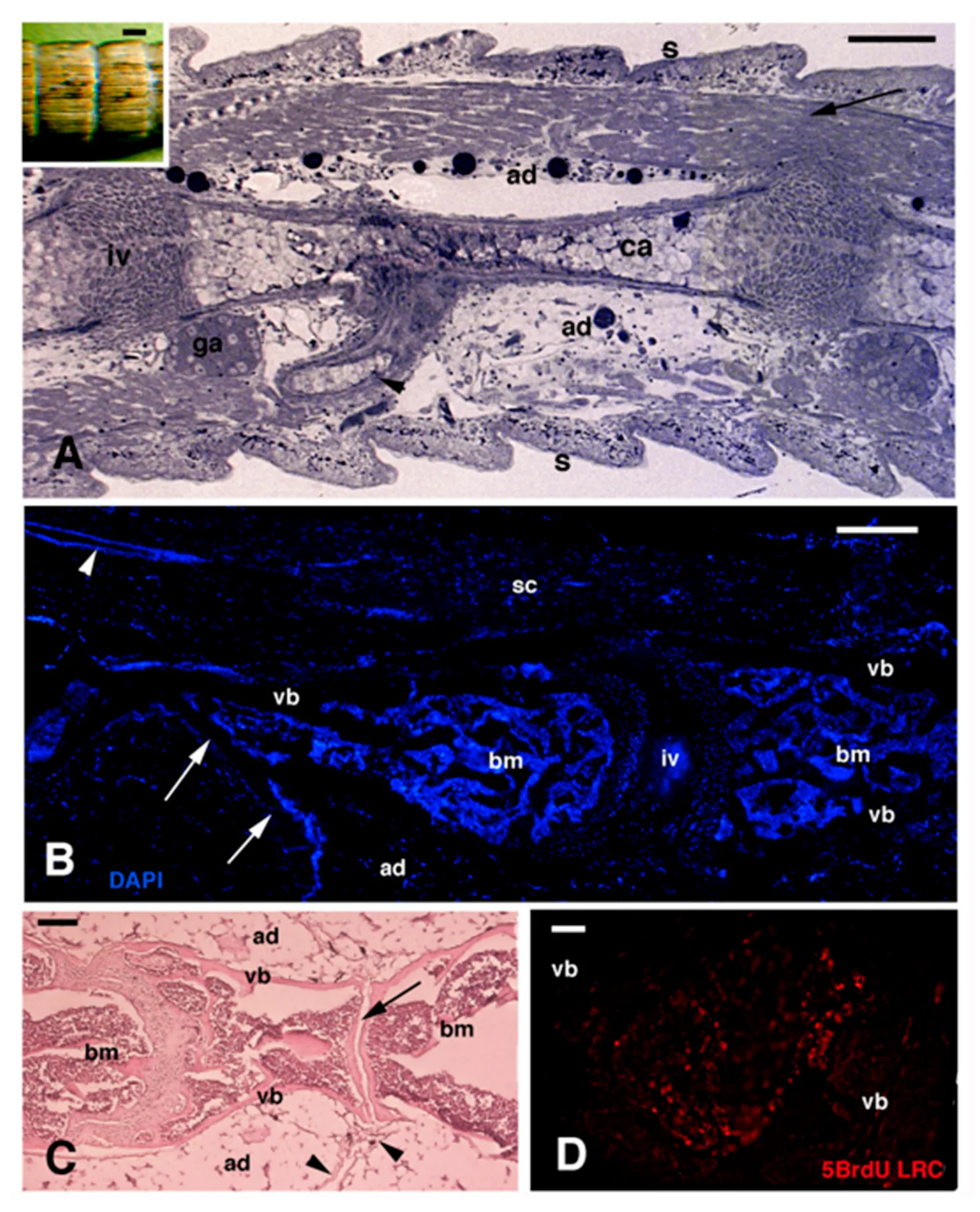
Normal tail histology in embryonic and adult tails of lizards (the caudal direction is on the right). (**A**) Longitudinal section of mid-tail level of an advanced embryo of *Anolis lineatopus* (stage 35–36, 7–10 days before hatching), showing the vertebral shape and the inserted intervertebral muscle fascicles (arrow). Most of the vertebra is made of cartilage, including the chevron bone (arrowhead). Toluidine blue stain; bar, 100 μm. The inset (bar, 0.5 mm) shows the regular circular scale rings of a normal tail in *Podarcis muralis*. (**B**) DAPI-fluorescence detail showing the cellular (nuclear) density in two adjacent vertebrae of *P. muralis*. The cell-rich bone marrow is evident in the trabecular bone, while the intra-vertebral fissure zone, in continuation with the connective autotomous plane, is partially detected (arrows). The arrowhead indicates the central canal and ependyma of the spinal cord; bar, 100 μm. (**C**) Frontal section of caudal vertebra in *P. sicula*, where the entire fission plane (arrow), dividing the vertebra in two parts, is evident. Arrowheads point to the inter-adipose connective autotomous plane joining the vertebral split. Hematoxylin-eosin stain; bar, 100 μm. (**D**) Immunofluorescence for LRCs (6 days 5BrdU-pulse and 4 weeks chase) within the bone marrow of a vertebra; bar, 10 μm. Legends: ad, adipose tissue; bm, bone marrow; ca, cartilage; ga, spinal ganglion; iv, intervertebral cartilage; mu, muscles (muscle bundle); s, scale; sc, spinal cord; vb, vertebral bone (compact).

In general biological terms, to fully grasp a biological phenomenon, the concept of evolution is the key. Evolution is not only referred to genes, anatomical and species modifications but also to biological processes present in these changing animals, including invertebrate and vertebrate regeneration [1,2,3,4,5,6]. Here I will deal with an example of a vertebrate that manifests regeneration as a result of an evolutionary process, the lizard. Among extant amniotes, a large process of heteromorphic and adaptive regeneration occurs in the tail of numerous species of lizards [7,8,9,10,11,12] (Figure 1). The key organ selected for the evolution of regeneration is the presence of a tail in these amniotes, made of numerous and integrated connective, vascular, skeletal, nervous, and epidermal tissues (Figure 2A–C). Aside from the more proximal 6–8 caudal vertebrae (pygopods vertebrae), most of the other distal caudal vertebrae present a narrow splitting fissure that divides the vertebrae into two pieces, forming an intra-vertebral fracture plane (Figure 2C). There is a correlation between autotomy and tail regeneration, two phenomena that possibly evolved together [8,9,13,14,15].

**Figure 3:**
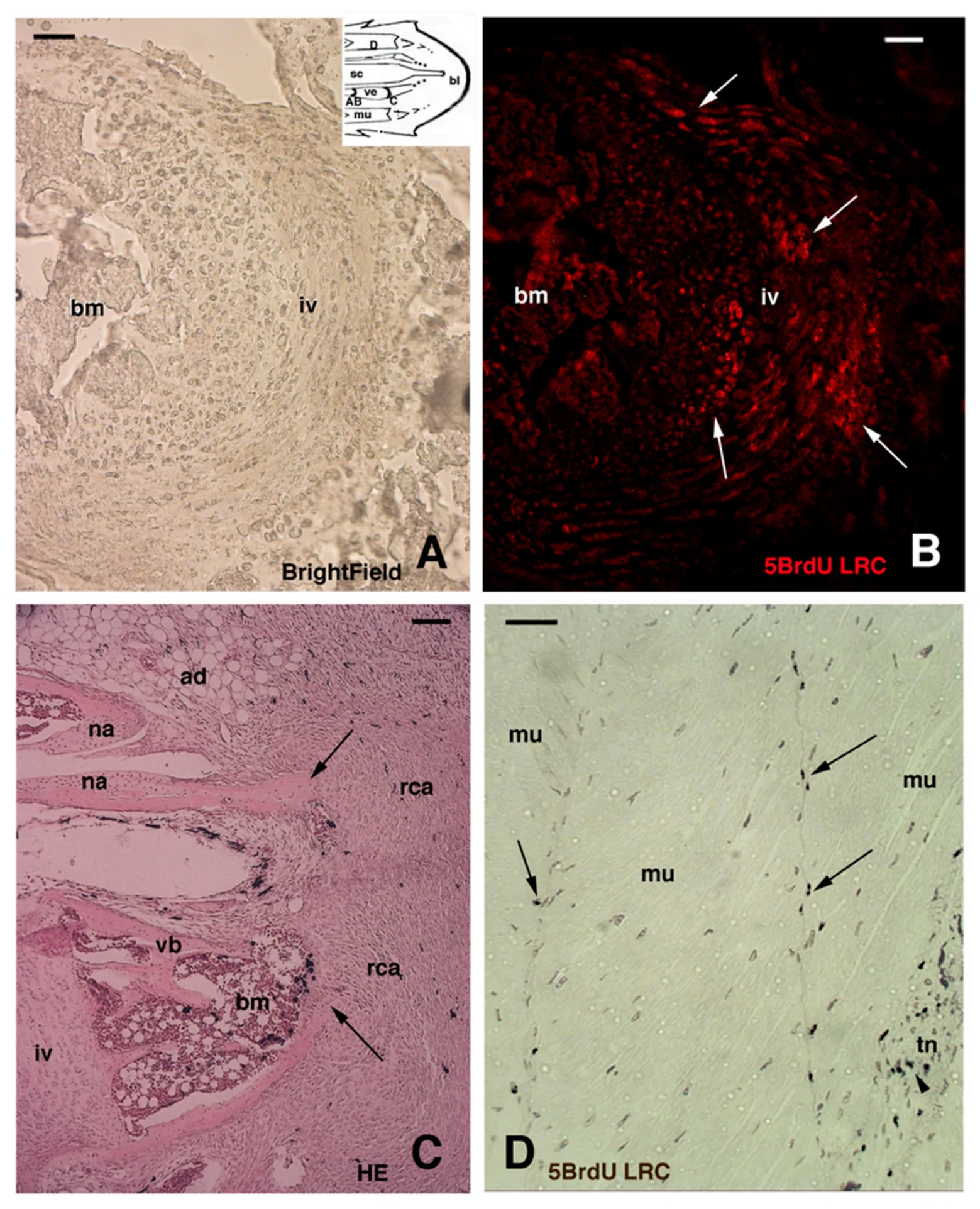
Histology and immunolabeling for 5BrdU-LRCs in stump tissues of *P. muralis*. (**A**) Bright-field close-up of intervertebral cartilage (cartilage between two vertebrae); bar, 20 μm. (**B**) Same image but observed under immunofluorescence for LRCs. Arrows indicate some 5BrdU-immunofluorescent cells; bar, 20 μm. (**C**) Region of transition between the autotomized vertebra (left) and the regenerated tail (right) at a cone stage. The bone tissues of the vertebra (arrows) are in continuation with cartilaginous cells of the regenerating tail. Hematoxylin-eosin stain; bar, 50 μm. (**D**) Detail on proximal stump muscles with LRCs (DAB-detection, dark nuclei) localized in the inter-muscle connective tissues (arrows) and tendons (arrowhead); bar, 20 μm. Legends: ad, adipose tissue; bm, bone marrow; iv, intervertebral cartilage; mu, muscles; na, neural arch; rca, regenerating cartilage; vb, vertebral bone.

**Figure 4:**
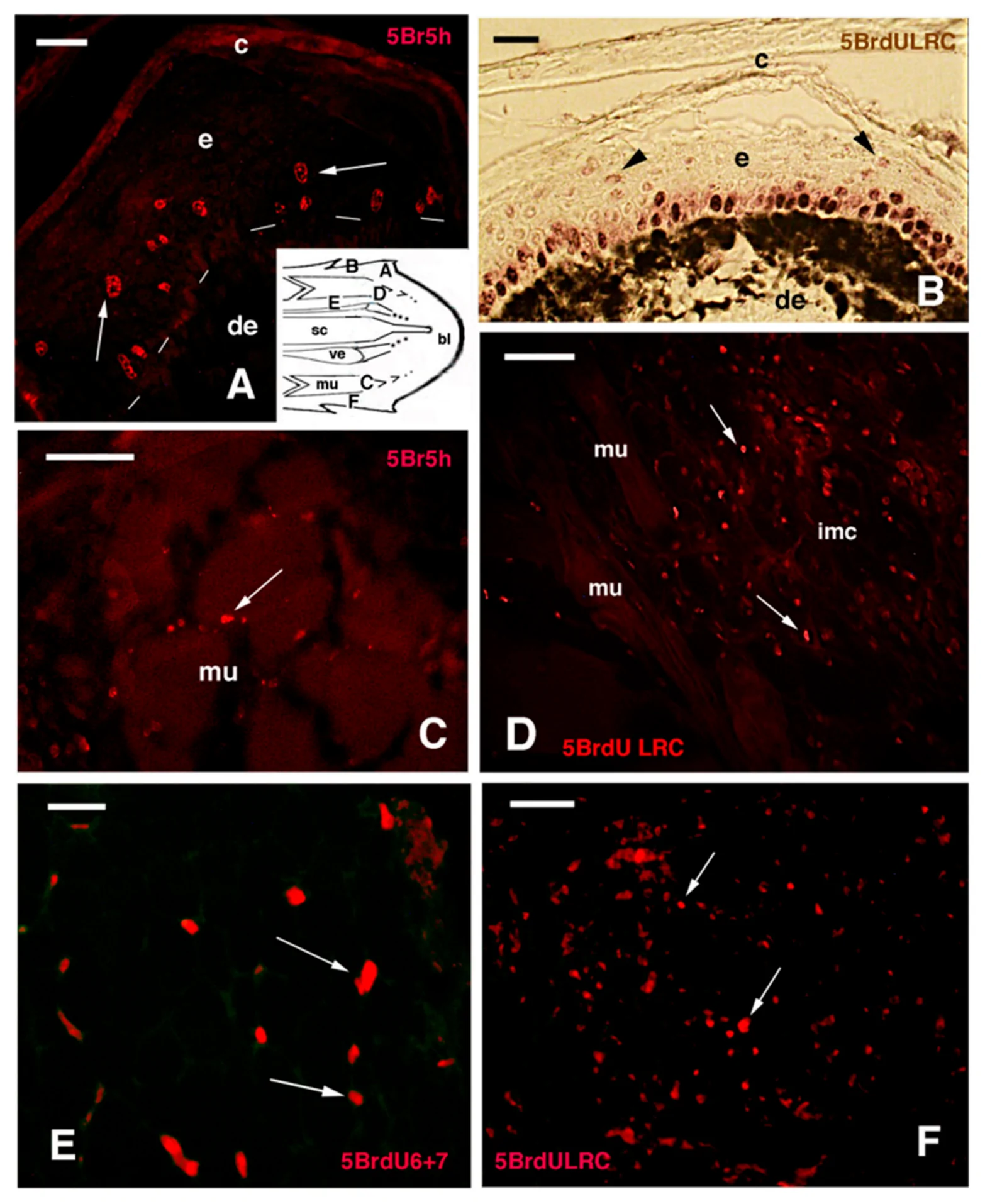
Immunohistochemical detection of 5BrdU-labeled cells 5 h post-injection (**A**,**C**), after a 6-day pulse and a 7-day chase (**E**), and for 5BrdU-LRC-labeled cells (**B**,**D**,**F**) in different stump tissues of *P. muralis*. (**A**) Sparse 5BrdU-immunolabeled nuclei (arrows) are seen in the epidermis of a stump scale close to the blastema-cone. Dashes underline the epidermis; bar, 10 μm. The inset drawing indicates the position of the following images. (**B**) Other proximal scale showing numerous LRCs (DAB-detection) in the basal and suprabasal (arrowheads) layers; bar, 10 μm. (**C**) Various 5BrdU-immunofluorescent nuclei (arrows, likely of satellite cells) associated with muscles of the stump are seen; bar, 20 μm. (**D**) Numerous LRCs (arrows) are present among the inter-muscle connective tissue located at the base of a blastema; bar, 20 μm. (**E**) Numerous 5BrdU-labeled nuclei (arrows) are seen in the peri-vertebral adipose tissue close to the blastema; bar, 10 μm. (**F**) Numerous LRCs (arrows) are seen in the dermis close to the blastema; bar, 20 μm. Legends: c, corneous layer; de, dermis; e, epidermis; imc, inter-muscle connective tissue; mu, muscles; sc, spinal cord; ve, vertebra.

**Figure 5:**
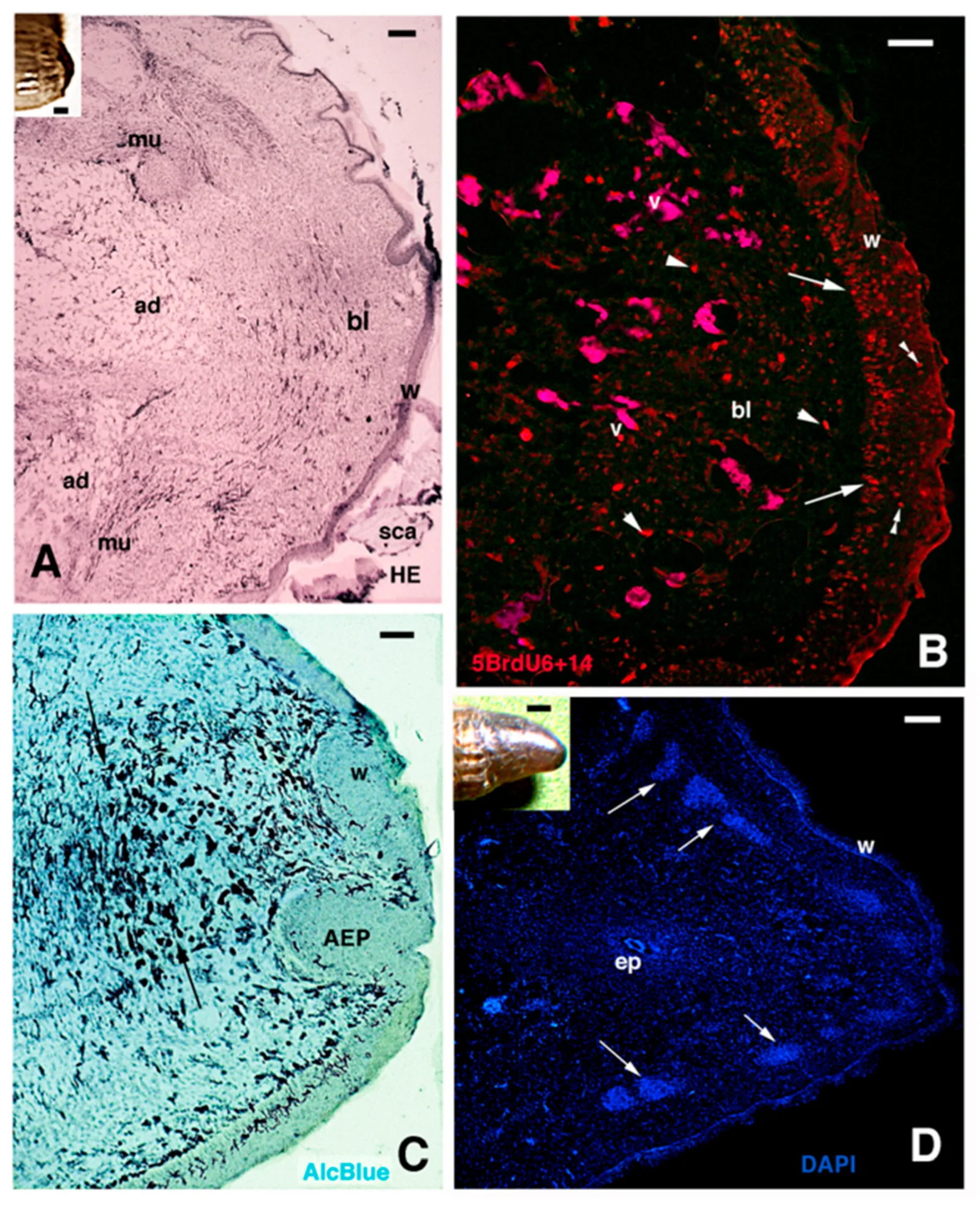
Histology of blastema (**A**,**B**) and blastema-cones (**C**,**D**) in *P. muralis*. (**A**) This section evidences the continuity between the mesenchymal blastema and stump tissues. Hematoxylin-eosin stain; bar, 50 μm. The inset (bar, 0.5 mm) shows an initial blastema. (**B**) 5BrdU-immunolabeled blastema cells (arrowheads), basal keratinocytes (arrows), and suprabasal cells (double arrowheads) in the wound epidermis after 6 days 5BrdU-pulse + 14 days chase. Pinkish autofluorescence of blood vessels; bar, 20 μm. (**C**) Apical blastema-cone showing a diffuse alcianophilic staining (indicating hyaluronate content). Arrows indicate the massive accumulation of melanophores; bar, 20 μm. (**D**) Regenerating cone featuring the repetitive regenerating myotomes (arrows). DAPI fluorescence; bar, 50 μm. The inset (bar, 1 mm) shows the gross aspect of a cone. Legends: ad, adipose tissue; AEP, Apical Epidermal Peg; bl, blastema; ep, ependyma; mu, stump muscles; v, blood vessels (autofluorescent); sca, scab fragments; w, wound epidermis.

**Figure 6:**
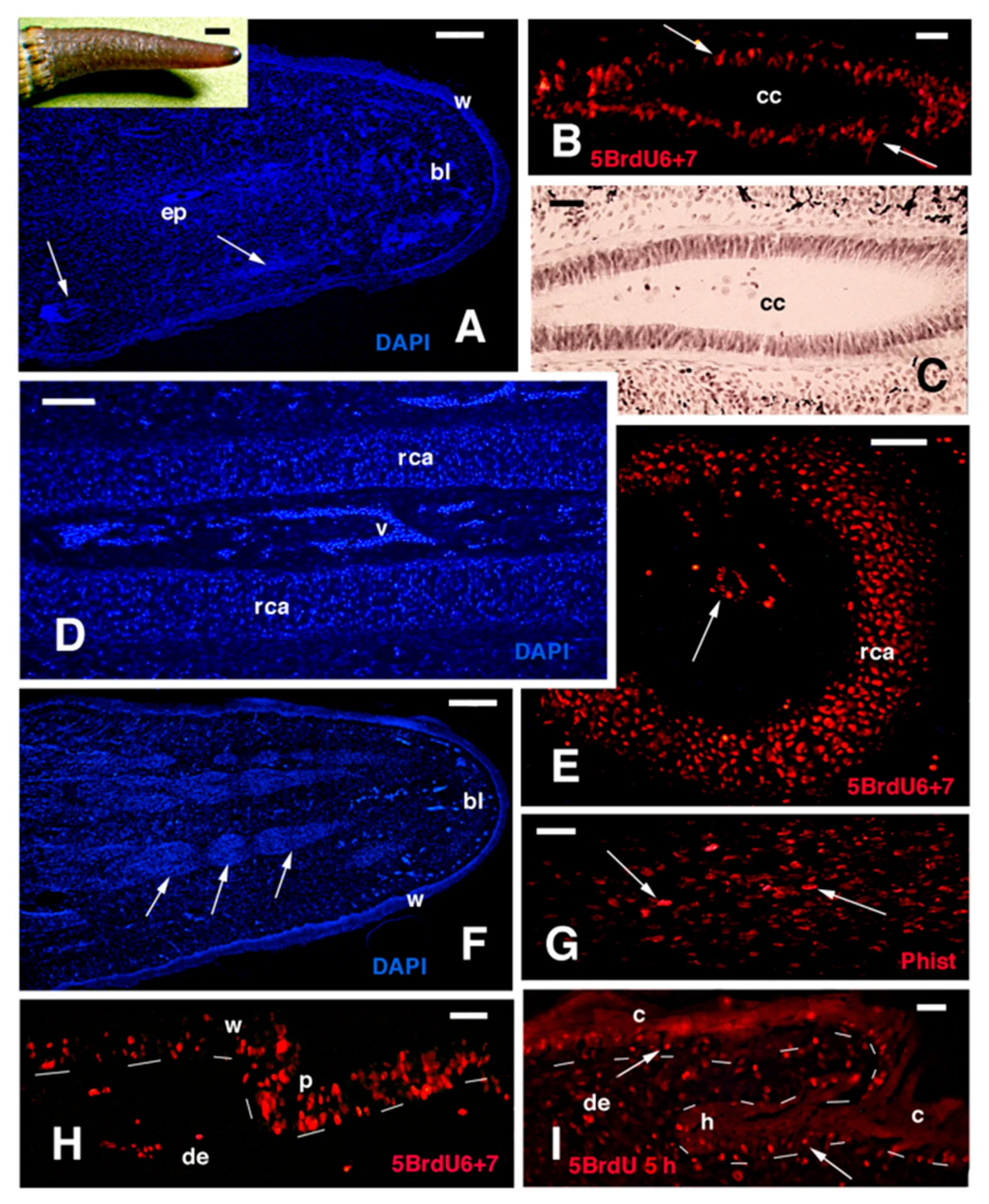
Histology and fluorescence of elongating (regenerating) cones in *P. muralis*. (**A**) DAPI fluorescence in the apical region of an elongating cone; bar, 100 μm. The inset (bar, 1 mm) shows the elongating (regenerating) tail. (**B**) Proliferating cells (arrows) in an ependymal ampulla after a 5BrdU-pulse of 6 days + 7 days chase; bar, 10 μm. (**C**), Epithelium of an ependymal ampulla stained with hematoxylin-eosin, surrounding the central canal; bar, 10 μm. (**D**) Longitudinal section of differentiated cartilaginous tube located at about 4 mm from the tip of the regenerating tail; bar, 50 μm. (**E**) Cross-sectioned cartilaginous tube located at about 3 mm from the tail tip with the thin central ependyma (arrow). Most cartilaginous cells are 5BrdU-labeled after 6 days of 5BrdU-pulse + 7 days of chase; bar, 50 μm. (**F**) Other tip of an elongating tail cut tangentially to show the myotomal organization (arrows) of the regenerating muscles. DAPI fluorescence; bar, 100 μm. (**G**) Detail of phospho-histone-labeled nuclei, indicating proliferating myoblasts localized within a regenerating muscle bundle; bar, 10 μm. (**H**) Numerous immuno-labeled nuclei are seen in wound epidermis folding into a peg after 6 days of 5BrdU-pulse + 7 days chase. Dashes underline the epidermis; bar, 10 μm. (**I**) Regenerating proximal scale with labeled nuclei (arrows) in the basal layer, 5 h after injection of 5BrdU. Dashes underline the epidermis; bar, 10 μm. Legends: bl, blastema (apex); c, corneous layer; cc, central canal; de, dermis; ep, ependyma; h, hinge region (inter-scale); p, epidermal peg; rca, regenerated cartilage; v, blood vessels (large vein); w, wound epidermis.

A strong muscle contraction can determine vertebral rupture, followed by detachment along the autotomous planes of the remaining tissues of the tail [16,17]. The formation of autotomous planes, surfaces of anatomical weakness traceable from caudal vertebrae to the skin, allows tail release under threatening/challenging encounters or after sizing the tail by a predator, while the tailless lizard can escape [13,14,15,16,17]. The present manuscript is based on experimental evidence provided in previous studies (examples are shown in Figure 2, Figure 3, Figure 4, Figure 5 and Figure 6), which are cited in the present MS [7,18,19,20]. It is from the connective tissues forming autotomous planes that some poorly differentiated cells, putative stem or progenitor cells, are likely localized, and these cells participate in the regeneration of the tail [18,19,20]. In comparison to tail cuts or injuries, autotomy determines little tissue damage and bleeding, and therefore limits inflammation, favoring tissue repair and regeneration.

How lizards evolved such an outstanding regenerative capability in their tails will likely remain unknown, but based on recent acquisitions, I here hypothesize a possible sequence in the evolution of tail regeneration as a process linked to tail autotomy. Lizards comprise about 5700 extant species, and their radiation initiated at the end of the Permian and continued in the mid-Triassic [21,22]. In spite of their generally small size, they represent the most successful forms of ectothermic reptiles, as is testified by the numerous species in comparison to the other reptilian groups, snakes, turtles, and crocodilians. A small size, however, indicates that lizards have been preyed upon since ancient times in the Permian and Triassic, and also today; they were tagged prey from other tetrapods, especially larger amniotes, sauropsids, or mammals. Due to low predation efficiency and survival from the Permian Period until today, these slender caudate reptiles must have possessed very successful mechanisms for escaping predators. The main avoidance mechanism is the presence of autotomous planes in their key organ, the tail [13,14,15]. Only by thinking in evolutionary terms can we get some explanation on how these anatomical planes of weakness originated in lizards in relation to regeneration (see later).

## 2. Lizards Form Regenerative Blastemas in Terrestrial Conditions

Inside tail vertebrae, an active hematopoietic bone marrow with localized blood long label-retaining cells (LRCs) is present. The reported data and Figure 2, Figure 3, Figure 4, Figure 5 and Figure 6 derive from experiments conducted in previous studies [18,19,20]. LRCs can be visualized by immunohistochemistry after injecting 5BrdU (5 Bromo deoxy uridine, a thymidine analog) for 3–5 h or with a longer treatment (e.g., 6 days 5BrdU-pulse + 5 weeks chase). They represent putative stem or progenitor cells from which blood cells are continuously formed (Figure 2D). Immature red and white blood cells formed in the marrow travel into the circulatory system and colonize the blastema as it forms, especially during the initial reparative phase. Aside from putative stem or progenitor cells from the hematopoietic marrow, other 5BrdU-LRCs, which are also telomerase-positive, likely represent transit amplifying cells. These putative stem or progenitor cells are localized in the intervertebral cartilage (Figure 3A,B) and in other tissues of the tail [18,19,20]. Immunolabeling for LRCs using 5BrdU has shown the presence of numerous labeled cells, putative stem cells, in the inter-muscle connectives and tendons joining muscle fascicles, including in the autotomous planes. It is from these connective planes that blastema cells are produced after an autotomous split of the tail (Figure 3C,D). In fact, these connective planes remain on the surface of the stump after autotomy. However, other putative stem cells (LRCs) have been observed in the epidermis, muscles (satellite cells), peri-vertebral connective and adipose tissues, and in the meninges surrounding the spinal cord (Figure 4A–F).

Initially, the regenerating tail blastema appears homogeneous, made of a loose connective tissue of mesenchymal cells and fibroblasts, with sparse proliferating cells immersed in an alcian blue-positive extracellular matrix containing hyaluronate [4]. Later, most of the blastema cells re-differentiate into the same type of cells migrated from stump tissues, although some cells may even trans-differentiate and give rise to other types of tissues [23]. The wound epidermis (regenerating) forms a stratified corneous layer made of soft keratins and lipid-rich corneocytes that impedes desiccation in aerial conditions and maintains a hydrated blastema that keeps growing by continuous cell proliferation into a new tail [4,9,24]. A problem with the liberation and proliferation of stem cells to form mesenchymal-like cells in the blastema is the potential negative action of the immune system. In fact, mesenchymal cells derived from stem cells or through de-differentiation from stump tissues may express embryonic antigens on their surface that interact with circulating adult immune cells. The latter cannot recognize these embryonic antigens, as they have never encountered them and have become tolerant to them due to the late maturation of adult immunocompetence [4,25,26]. Therefore, together with a mechanism of regulation of cell proliferation, lizards also evolved a mechanism of immune suppression to “protect” blastema cells from the attack by definitive/adult lymphocytes and macrophages. At the same time, the proliferation of blastema cells and wound keratinocytes remains regulated and is not detoured toward a tumorigenic growth.

After the blastema assumes a coniform shape, peripheral segmental muscles (myotomes) and a central ependymal epithelium surrounded by a continuous, axial cartilaginous cylinder gradually differentiate as the forming tail elongates (Figure 1B,F,H, Figure 5D and Figure 6A). Although the fully regenerated tail also contains numerous, integrated tissues, it lacks a segmented vertebral column, which is replaced by an unsegmented cartilaginous cylinder, and the spinal cord, with sensory and motor neurons and spinal ganglia, is replaced with a narrow ependymal tube. During tail elongation (Figure 1B,F,G,H) intense cell proliferation takes place in early differentiating tissues such as the ependyma (Figure 6B,C), the cartilaginous tube (Figure 6D,E), the new muscles (Figure 6F,G), and the forming new scales (Figure 1H,I). A large mass of new muscles is regenerated in the tail, but these muscles are segmentally arranged (Figure 5D and Figure 6F, see later). These lepidosaurians cannot regenerate other body appendages or organs as in the tail, although they also possess a remarkable regeneration of large masses of cartilage and muscles [4,10,11,12].

## 3. Hypothesis on the Evolution of Autotomy and Regeneration in Lizards

Although tail autotomy is a typical characteristic of most lizards, this phenomenon is also present in some caudate amphibians [27,28]. Some extant urodeles (plethodont salamanders) develop autotomous planes in their tails, from where the tail is regenerated after loss [27,29,30,31]. Regenerated tails are frequently detected in extant plethodont amphibians, common in *Hydromantes* [28], and variable in different species of American plethodonts (4.9 to 23% [27]). Like in lizards, also in salamanders, regeneration can initiate after autotomy or after a cut that determines tail amputation [7,29,30]. This behavior, dropping the tail under predator challenge, was likely also present in ancient salamanders of the Carboniferous that later regenerated the tail. Regeneration after amputation that damages various tissues, not from autotomous planes, may indicate that extant salamanders and lizards possess tissue-distributed stem cells, and not exclusively localized in autotomous planes. This is seen after studying the tissue distribution of 5BrdU-LRCs, many of which are still found in inter-muscle connective septa, suggesting that blastema cells can produce new stem elements after a regeneration event [18].

**Figure 7:**
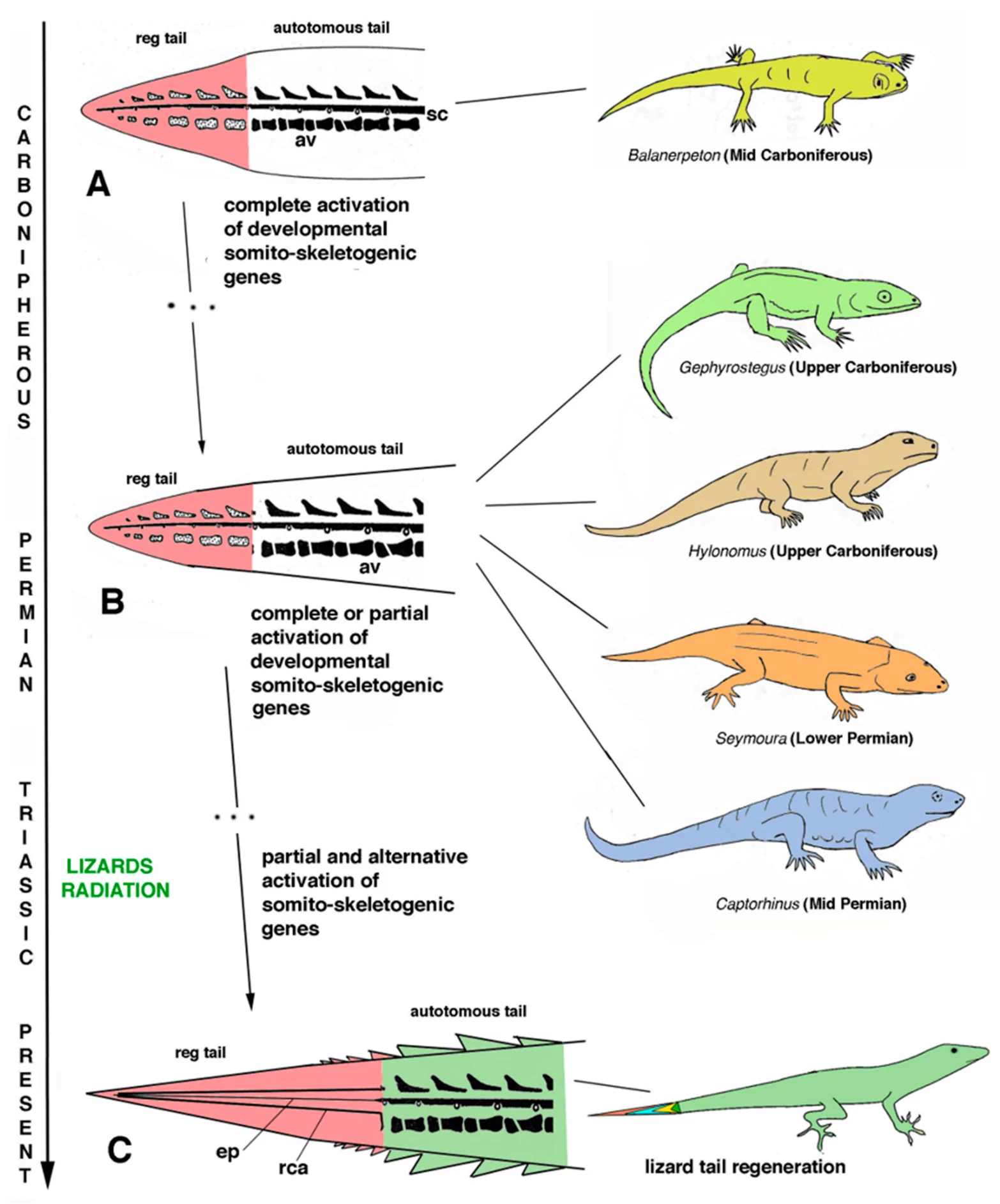
Hypothetical representation of the evolution of caudate amphibians into caudate reptiles during progressive geological periods (vertical arrow on the left). The hypothesized autotomous tails present in caudate amphibian ancestors living in the Carboniferous (**A**) were “inherited” in successive reptiliamorphs living in the Upper Carboniferous–Permian (**B**), and “conserved” in modern lizards that radiated from the Triassic to the present time (**C**). Original vertebrae in black; regenerating vertebrae are stippled. Legends: av, autotomous vertebrae; ep, ependyma; rca, regenerated cartilaginous tube; sc, spinal cord. See text for explanation.

Based on the above information, I submit that tail regeneration was also present in ancient amphibians, and this hypothesis is currently supported by fossilized remnants, including the lepidospondilous *Microbrachis pelikani* and other fossilized amphibians [31]. I therefore hypothesize that autotomous planes were already present in the tail of anthracosaur ancestors of amniotes, shifting back the origin of autotomy and regeneration from reptiles to Paleozoic caudate amphibians, perhaps resembling salamanders such as *Balanerpeton* or *Microbrachis* (Figure 7A). The present hypothesis suggests that reptiliamorph basal amniotes inherited autotomous tails from amphibians in the late Carboniferous–Early Permian (exemplified in *Gephyrostegus* and *Hyolomus* in Figure 7B), then in *Seymouria* anthracosaurs in the Lower Permian (Figure 7B), later in captorhinomorphs in the Middle–Upper Permian such as *Captorhinus* (Figure 6B) and, finally, in true lizards of the Triassic (Figure 7C). Also, these lepidosaurians, like ancient caudate anthracosaurs, were able to regenerate their tails, although heteromorphic (see later). It is submitted that the presence of tails that can break provided a selective advantage to these ancient lepidosaurians for their survival during the long geological periods up to the present time. Without this self-defense mechanism, most species of lizards would likely be much more reduced in numbers, even extinct, and not as numerous as in the present time.

Since the beginning of their evolutionary story, from the Upper Carboniferous–Lower Permian in captorhynomorphs, during the Permian in eosuchians, and later in the Triassic lizards, these slender reptiles likely possessed long tails that already contained autotomous planes [32,33,34,35] (Figure 7). This key organ, the tail, is important for numerous reasons [13,14,15], but is not immediately essential for survival. Therefore, tail loss was subjected to positive selection for regeneration initially in ancestral amphibians, perhaps by epigenetic mechanisms that made regeneration of the tail advantageous. Therefore, the origin of autotomous planes can now be moved from reptiles to the development of the tail in ancient amphibians, and this can also be analyzed in extant amphibians with autotomous tails. As previously discussed, regeneration in amphibians derived from their life cycles that included developmental genes operating during larval stages and metamorphosis, which were reutilized for regeneration [4,36]. The importance of the tail for balance and locomotion in terrestrial conditions is high, and regenerating the tail was therefore an advantageous character for semi-aquatic anthracosaurian amphibians. The histological study on the formation of autotomous tails in extant urodeles would be essential for envisioning the process that also occurred/occurs in tailed basal amniotes and lepidosaurians [37,38].

The present hypothesis is supported by some fossils [31], showing that tail regeneration was present since the transition between amphibians and reptiles and that the two processes were long developed in eosuchian progenitors of lepidosauriamorphs in the Upper Carboniferous–Permian [32,33,34,35] (Figure 7B). These quadrupedal reptiliamorphs possessed elongated bodies, short limbs, and a variably long tail. In their physical reconstructions, these ancient and small- to medium-sized amphibians and reptiles (30–60 cm in length) are represented with lizard-like features such as *Gephyrostegus* (Upper Carboniferous), *Hylomonus* (Upper Carboniferous), *Seymouria* (Permian), or *Captorhinus* (Permian) (Figure 7A,B). In conclusion, it is hypothesized here that autotomy and regeneration were ancestral characters present in lepidosaurians and that they were maintained in most lizard families (Figure 7C). Autotomy and regeneration were possibly lost during the Mesozoic only in a few families of lizards (mainly varanids, helodermatids, chamaleontids, and numerous agamids), probably in relation to specific ecological adaptations or the loss of predatory challenge, as amply discussed in previous studies [13,14,15].

## 4. The Regenerated Tail of Lizards Derives from Alternative Gene Activities

In previous studies, it was hypothesized that during the formation of autotomous planes in the developing tail of lizard embryos [37,38], some putative stem or progenitor cells were distributed inside these planes during the peculiar somite fusion that gives rise to autotomous planes [9]. This hypothesis is indirectly supported by the results that follow the ablation of the tail bud in lizards during early embryogenesis, an operation that is not followed by tail regeneration [39,40]. It was suggested that the removal of the tail bud basically eliminates the entire pre-somitic mesoderm and the incorporated putative stem or progenitor cells, preventing development and regeneration of the embryonic tail [9]. It is here submitted that during their land adaptation, lizard ancestors, such as captorhinomorphs and eosuchians, already possessed anatomical planes of fracture in the tails passing through the vertebrae, neural arches, inter-muscle septa and terminating underneath the epidermis of scales, as also supported by some fossils [8,32,33,34,35] (Figure 7B). Possibly, due to some gene changes/mutations that eosuchian diapsids underwent, an alteration of the morphogenetic process of segmentation of their caudal vertebrae during sclerotome formation occurred, and this modification gave rise to split tail vertebrae, like in extant lizards [37,38]. This process might have occurred through the alteration of some developmental somitic-skeletogenic genes or of their timing of expression within the developmental sequence, leading to the morphogenetic process of inter-segmental vertebrae formation [41,42,43].

After the loss of larval stages and metamorphosis from their amphibian ancestors during land adaptation in the Upper Carboniferous, amniotes also lost a large part of the regenerative ability present in their amphibian progenitors [4,6,36]. In particular, a large part of developmental somitic-skeletogenic genes were lost or were altered in their expression, limiting the regeneration of a functional tail like the original. However, somehow these ancient lepidosaurians were able to evolve an alternative developmental process for regenerating numerous tissues, although incapable of forming a segmented tail like the original. Alternative morphogenetic processes for regenerating the tail are even present in some urodeles, considered the “best vertebrate regenerators”. In fact, whereas during development caudal vertebrae derive from the colonization and replacement of the notochord by sclerotomic cells, during tail regeneration the new vertebrae are formed from the sculpturing of an initial rod of cartilage [7,29,30,44,45]. This observation indicates that an exact repetition of the morphogenetic process is not possible even in these amphibians, suggesting that tail regeneration occurs through an alternative sequence of developmental gene activation, different from that operating during embryonic development.

Importantly, such a hypothetical regenerative process in reptiliamorphs involves the activation of oncogenes and tumor suppressors, like in extant lizards, indicating that reptilian tail regeneration also involved acquiring some anti-cancer mechanisms previously present in their amphibian ancestors [4,25,46,47]. The latter mechanisms, largely unknown in molecular terms, prevent a neoplastic transformation from taking place during tail regeneration.

## 5. How Lizards Might Have Evolved Alternative Developmental Gene Pathways

**Figure 8:**
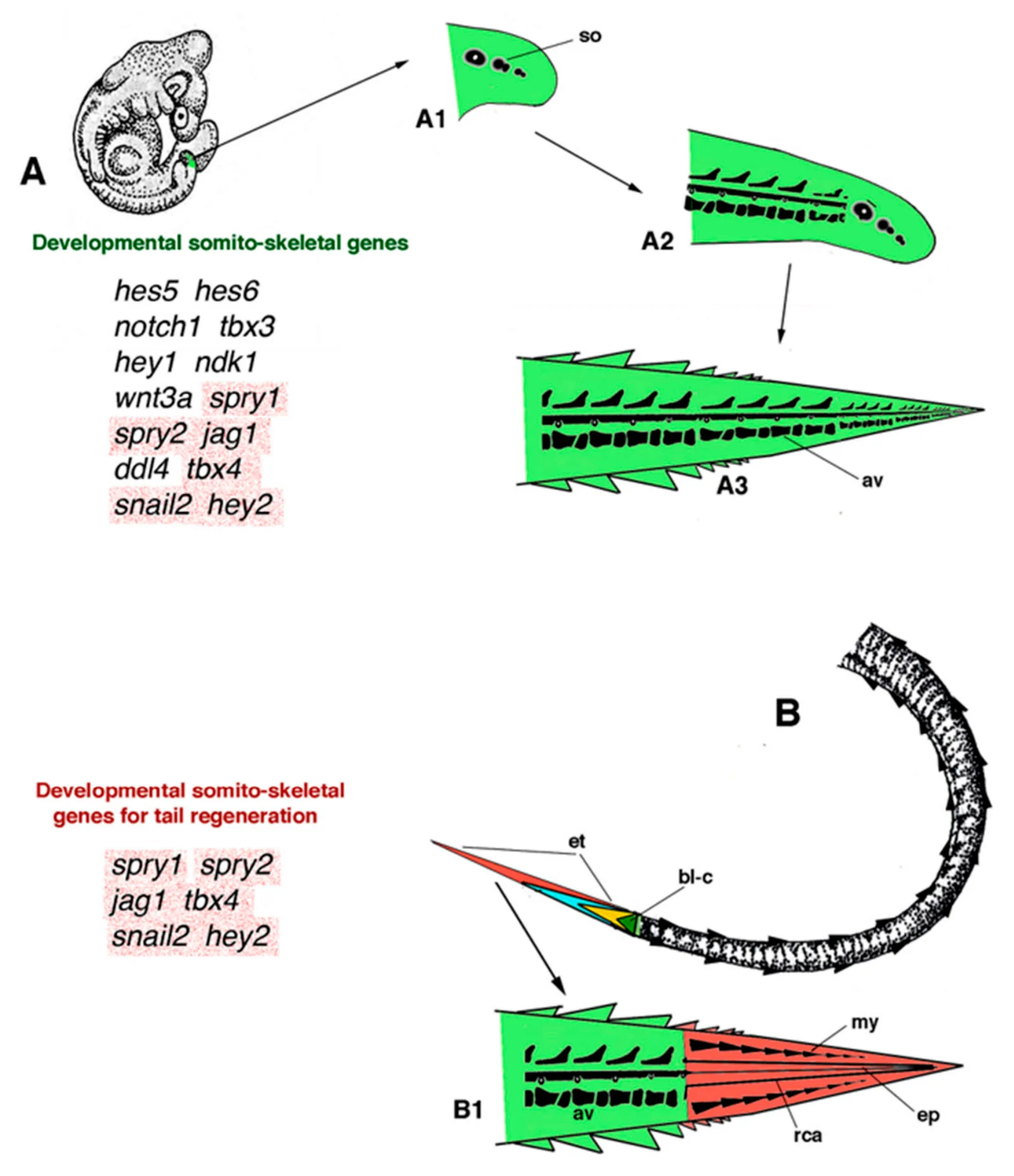
Schematic drawings featuring some of the main somitic-skeletogenic genes involved in vertebrae morphogenesis, activated during tail development (**A**) and during tail regeneration (**B**). (**A**) Tail formation from the tail bud (A1) and growth (A2–A3), during embryonic development with the morphogenesis of caudal vertebrae. A1, tail bud with forming somites; A2, elongating tail bud with formed proximal vertebrae and somites in more distal regions; A3, adult tail with autotomous vertebrae (in black). (**B**) Regenerating tail with progressive stages of regeneration (evidenced with different colors), where fewer genes are activated in comparison to the embryo. B1, detail of normal (stump; green) with autotomous vertebrae, and regenerated (red) tail containing a continuous cartilaginous tube and regenerating myotomes. Legends: av, autotomous vertebrae; bl-c, blastema-cone; et, elongating regenerating tail; my, myotomes (regenerated); so, somites; See text for explanation.

Molecular analysis of gene activation during regeneration has allowed the discovery of the main genes activated during the process [48,49,50,51,52,53,54]. Recent studies have revealed that the regenerating tail is formed from the activation of alternative gene pathways with respect to those utilized during the development of the tail [36,43,49,50]. Tail regeneration does not activate the same genes, and in the same sequences as during tail development, since, as indicated in the introduction, regeneration initiates from a multicellular body and not from a zygote. In particular, the recent study on the Tokay gecko (*Gekko gecko*) allows detecting the differences in expression of developmental genes versus those expressed during tail regeneration [43]. No unique genes for regeneration are utilized, but only some of the developmental genes initially activated, likely in a different and reduced pattern of gene activation (alternative developmental gene pathways; Figure 8A,B).

**Figure 9:**
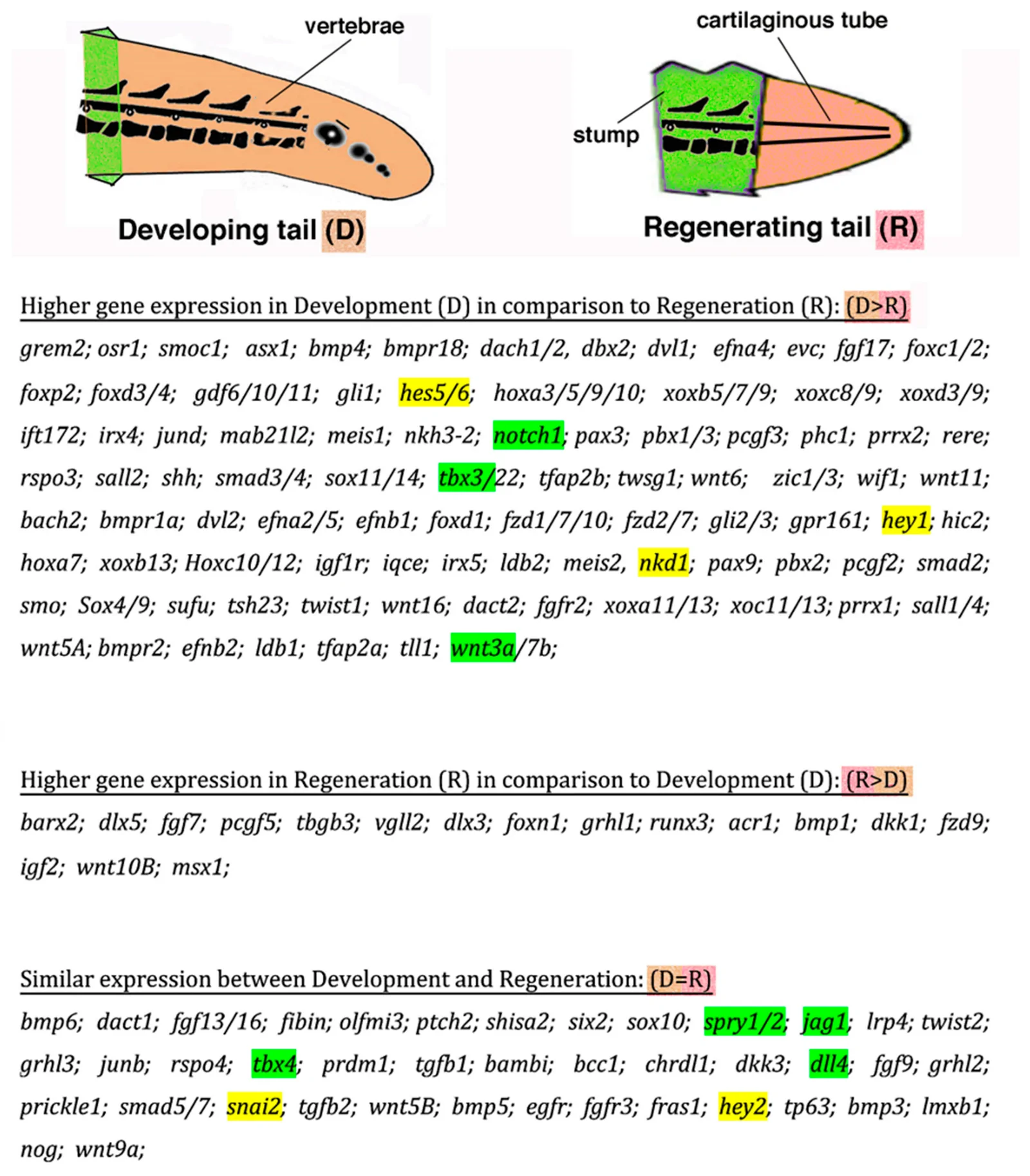
Same toolkit developmental genes (D, 16 days of embryonic tail development) versus the same genes utilized for regeneration (R, 28 days tail regeneration) in *Gekko gecko* (see Figure 1F in [43]). Somitogenic genes (see Table 1 in Eckalbar et al., 2012 [41]) are highlighted in green, and include oscillatory genes that are specifically highlighted in yellow. A higher number of genes (upper list) are more up-regulated during development than during regeneration (D > R), and a small number of genes (short intermediate list) are more up-regulated in regeneration in comparison to development (R > D). An intermediate number of up-regulated genes (bottom longer list) shows a similar expression between tail development and regeneration (D = R).

In order to point out the difference in some gene expression between development and regeneration (Figure 8 and Figure 9), here we only indicate few examples derived from the comparison between common genes expressed in the Tokay gecko embryos at 16 days of development (with an elongating and curled tail) with those expressed in the developing tail at 28 days of regeneration (elongating cone; see [43], especially their Figure 1). At these stages, the developing tail (D) contains various differentiating tissues, and the regenerating tail (R) also contains numerous differentiating tissues. Also, we consider only key development genes (toolkit genes) with higher differences in their expression, as reported in the heatmap in Figure 1F of [43]. While the developing tail expresses hox genes (*xox a*,*b*,*c*,*d*) in a sequence in agreement with their chromosome localization, this does not occur in the regenerating tail [43]. Other differences in gene expression, D > R, R > D, D = R, are reported in Figure 9, which only gives an indicative perception of the difference in activity for these genes. Furthermore, these studies have also shown that a number of genes activated during tail development are not activated during tail regeneration (*hes1*, *mam13*, *idi1*, *idi2*, *cyr61*, *nkd2*, *tcf712*, *glcci1*, *snail2*, *ctgf*, *nuoak1*, *hhex*, *slc2a3*, *klf10*, *bhlhb2*, *amotl2*, *rasa4*, *znf537*, *c14orf43*, *axud1*, *smurf2*). The different pattern of expression for individual genes, or for genes interacting with other genes, eventually determines the formation of a higher anatomical complexity in the developing tail compared with the lower complexity of the regenerating tail.

Despite the lack of activation of the developmental genes that were utilized during embryogenesis of the tail, lizards can, however, regenerate a heteromorphic, large tail using most of the same genes, but with different timing, sequence, interactions, and intensity of activation (Figure 9). The molecular mechanism of selection of developmental genes utilized from blastema cells of different tissue origin to rebuild a new heterogenous tail is unknown, but it likely operates after the accumulation over the stump tissues of a mass of heterogenous cells of different tissue origin, forming the blastema. Within this mass, some cells of myogenic origin initiate reactivation of genes for myogenic differentiation, other cells of chondro-osteogenic derivation reactivate genes for chondrogenic differentiation, others mesenchymal cells turn on genes for connective or adipose differentiation, etc. [23,48,55,56]. As previously indicated, a blastema formed in an adult body is not equivalent to a single fertilized egg that gives rise to an embryo and, consequently, it is impossible that all myogenic, chondrogenic, neural, etc., genes are reactivated with the same pattern as during development.

The study on the Tokay gecko also indicates that many, or even fewer, genes are expressed in the embryonic tail at 16 days in comparison to the regenerating tail at 28 days (see Figure 1C–F in [43]). In Figure 7 and Figure 8, only some important genes (toolkit genes) are reported. Therefore, referring only to commonly expressed genes, the principal differences between the developing and the regenerating tail regard genes that are involved in stemness, connective tissue formation, HOX genes for compartmentalization, genes for segmentation, immunocompetence, myogenesis, and neural formation. This study on a gecko lizard clearly shows that, even for commonly expressed genes, large differences in expression are present between development and regeneration of the tail. I stress again that differences in gene activation indicate that in the lost tail stump of lizards, formed from millions of cells, the reactivation of the same developmental programs of tail embryogenesis is impossible, especially in the terrestrial conditions that make organ regeneration very difficult [4,36].

From molecular studies, it is known that the lack or experimental ablation of some genes (knock-out or CRISPcas9 inactivation) can be partially or fully buffered by the co-option/co-activation of vicarious/compensation genes with similar roles (gene redundancy), so that the mutation is not manifested [57]. This suggests that the genome and its functional regulation in animals, perhaps under epigenetic modification or gene alteration, can utilize alternative developmental gene pathways during evolution when these changes do not determine abnormalities or death. This phenomenon indicates that the regenerating tissues derive from a different selection and activation intensity of similar or unique genes. Lizards likely evolved alternative developmental pathways for regenerating the tail that inevitably produced heteromorphic, deficient new tails. The latter, however, served to enhance survival, since the new tail was/is very useful and important for balance and running and is even improved compared to the original tail for fat storage and energy consumption [8,13,14,15].

## 6. Conclusions

In the case of lizards (and amphibians), other genes activated during tail development and absent or differentially activated for tail regeneration will very likely be discovered in future studies. This demanding molecular work is in the hands of future generations of biologists if we want to grasp the process and translate, at least in part, this knowledge to other amniotes in an attempt to improve their regenerative ability. The present discussion clearly stresses that, since development and regeneration begin from different cellular aggregations, a zygote vs. a mass of billions of somatic cells, gene activation and morphogenesis proceed with different modalities in the two cases. Consequently, regenerated organs are formed by a different pattern of gene expression and histogenesis from the original developmental program, and this is valid for any invertebrate or vertebrate, independently of its ability to regenerate [5].

In conclusion, as anticipated at the beginning of the present paper, the speculations submitted in this paper indicate that the phenomenon of tail regeneration in lizards, like for other cases of animal regeneration, derived from the specific conditions present at the beginning of their evolution. The present hypothesis suggests that the autotomous tail and tail regeneration present in salamander-like, caudate amphibian ancestors were inherited from Paleozoic lepidosaurians and later from lizards. Hypothetical epigenetic changes that took place during the evolution of autotomy and regeneration in ancient amphibians were likely driven by their amphibious lifestyle and environmental interactions. The study of the development of autotomy in caudate amphibians, in comparison to that present in autotomous lizards, will provide further information on the cellular origin of tail autotomy and regeneration. The process produced heteromorphic regenerated tails during terrestrial adaptation, missing a large part of the somitic-skeletogenic genes and the derived vertebral column, spinal cord, and precise skeletal-muscle insertions. Despite these shortcomings, regenerated tails have allowed the survival of numerous species of lizards to the present. The information on alternative developmental gene pathways will be useful for planning future attempts to stimulate organ regeneration in other amniotes, including humans.

## Data Availability

No new data were created or analyzed in this study. Data sharing is not applicable to this article.

## References

[1] Tiozzo S., Copley R.R. (2015). Reconsidering regeneration in metazoans: An evo-devo approach. Front. Ecol. Evol..

[2] Ricci L., Srivastava M. (2018). Wound-induced cell proliferation during animal regeneration. Wiley Intrdisci. Rev. Dev. Biol..

[3] Elchaninov A., Sukhikh G., Fatkhudinov T. (2021). Evolution of regeneration in animals: A tangled story. Front. Ecol. Evol..

[4] Alibardi L. (2021). Review. Tail regeneration in lepidosauria as an exception to the generalized lack of organ regeneration in amniotes. J. Exp. Zool..

[5] Alibardi L. (2024). Speculations on the loss of regeneration derived from developmental modifications during land adaptation in some evolutionary lineages of animals. Acta Zool..

[6] Zaraisky A., Araslanova K.R., Shitikov A.D., Tereshina M.B. (2024). Loss of the ability to regenerate body appendages in vertebrates: From side effects of evolutionary innovations to gene loss. Biol. Rev. Camb. Philos. Soc..

[7] Alibardi L., Sala M. (1983). Distribuzione di sostanze d’ importanza morfogenetica in tessuti rigeneranti di *Lacerta sicula*, *Triturus alpestris* e *Rana dalmatina*. Atti Mem. Accad. Patav. Sci. Lett. Arti.

[8] Bellairs A.d’A., Bryant S.V., Gans C., Billet F., Maderson P.F.A. (1985). Autotomy and regeneration in reptiles. Biology of the Reptilia.

[9] Alibardi L. (2010). Morphological and Cellular Aspects of Tail and Limb Regeneration in Lizard: A Model System with Implications for Tissue Regeneration in Mammals.

[10] Fisher R.E., Geiger L.A., Stroik L.K., Hutchins E.D., George R.M., DeNardo D.F., Kusumi K., Rawls J.A., Wilson-Rawls J. (2012). A histological comparison of the original and regenerated tail in the green anole, *Anolis carolinensis*. Anat. Rec..

[11] Lozito T.P., Tuan S.R. (2016). Lizard tail regeneration as an instructive model of enhanced healing capabilities in an adult amniote. Connect. Tiss. Res..

[12] Jacyniak K., McDonald R.P., Vickaryous M.K. (2017). Tail regeneration and other phenomena of wound healing and tissue restoration in lizards. J. Exp. Biol..

[13] Arnold N. (1984). Evolutionary aspects of tail shedding in lizards and their relatives. J. Natur. Hist..

[14] Arnold N. (1990). The throwaway tail. New Sci..

[15] Barr J.I., Boisvert C.A., Bateman P.W. (2021). At what cost? Trade-off and influence on energetic investment in tail regeneration in lizards following autotomy. J. Dev. Biol..

[16] Quattrini D. (1954). Piano di autotomia e rigenerazione della coda nei Sauri. Arch. Ital. Di Anat. Ed Embriol..

[17] Ritzman T.B., Stroik L.K., Julik E., Hutchins E.D., Lasku E., DeNardo D.F., Wilson-Rawls J., Rawls A.J., Kusumi K., Fisher R.E. (2012). The gross anatomy of the original and regenerated tail in the green anole (*Anolis carolinensis*). Anat. Rec..

[18] Alibardi L. (2015). Immunolocalization indicates that both original and regenerated lizard tail tissues contain populations of long retaining cells, putative stem/progenitor cells. Microsc. Res. Tech..

[19] Alibardi L. (2015). Original and regenerating lizard tail cartilage contain putative resident stem/progenitor cells. Micron.

[20] Alibardi L. (2019). Temporal distribution of 5BrdU-labeled cells suggests that most injured tissues contribute proliferating cells for the regeneration of the tail and limb in lizard. Acta Zool..

[21] Evans S.E. (2003). At the feet of the dinosaurs: The early history and radiation of lizards. Biol. Rev..

[22] Benton M.J. (2025). Vertebrate Paleontology.

[23] Londono R., Wenzhong W., Wang B., Tuan R.S., Lozito T.P. (2017). Cartilage and muscle cell fate and origin during lizard tail regeneration. Front. Bioengen. Biotechn..

[24] Maderson P.F.A., Roth S.I. (1972). A histological study of the early stages of cutaneous wound healing in lizards in vivo and in vitro. J. Exp. Zool..

[25] Alibardi L. (2017). Review. Biological and molecular differences between tail regeneration and limb scarring in lizards: An inspiring model addressing limb regeneration in amniotes. J. Exp. Zool..

[26] He B., Song H., Wang Y. (2021). Self-control of inflammation during tail regeneration of lizards. J. Dev. Biol..

[27] Wake D.B., Dresden I.G. (1967). Functional morphology and evolution of tail autotomy in salamanders. J. Morphol..

[28] Romano A., Amat F., Rivera X., Sotgiu G., Carranza S. (2010). Evidence of tail autotomy in the European plethodontid *Hydromantes* (*Atylodes*) *genei* (Temmick and Schlegel, 1838) (Amphibia: Urodela: Plethodontidae). Acta Herpethol..

[29] Mufty S.A., Simpson S.B. (1972). Tail regeneration following autotomy in the adult salamander, *Desmognatus fuscus*. J. Morphol..

[30] Dinsmore C.E. (1979). Tail regeneration in the plethodontid salamander, *Plethodon cinereus*: Induced autotomy versus surgical amputation. J. Exp. Zool..

[31] van der Voss W., Witzmann F., Frobisch N.B. (2018). Tail regeneration in the Paleozoic tetrapod *Microbrachis pelikani* and comparison with extant salamanders and squamates. J. Zool..

[32] Price L.I. (1940). Autotomy of the Tail in Permian Reptiles. Copeia.

[33] Dilkes D.W., Reisz R.R. (1986). The axial skeleton of the Early Permian reptile *Eocaptorhinus laticeps* (Williston). Canad. J. Earth Sci..

[34] Delfino M., Sánchez-Villagra M.R. (2010). A survey of the rock record of reptilian ontogeny. Semin. Cell Dev. Biol..

[35] LeBlanc A.R.H., McDougall M.J., Haridy Y., Scott D., Reisz R.R. (2018). Caudal autotomy as anti-predatory behaviour in Palaeozoic reptiles. Sci. Rep..

[36] Alibardi L. (2020). Appendage regeneration in anamniotes utilizes genes active during larval-metamorphic stages that have been lost or altered in amniotes: The case for studying lizard tail regeneration. J. Morphol..

[37] Werner Y.L. (1971). The ontogenic development of the vertebrae in some gekkonid lizards. J. Morphol..

[38] Winchester L.W., Bellairs A.D.A. (1977). Aspects of vertebral development in lizards and snakes. J. Zool..

[39] Marcucci E. (1915). Gli arti e la coda della *Lacerta muralis* rigenerano nello stadio embrionale?. Bollett. Soc. Natur. Napoli.

[40] Moffat L.A., Bellairs A.D. (1964). The regenerative capacity of the tail in embryonic and post-natal lizards (*Lacerta vivipara* Jacquin). J. Embryol. Exp. Morphol..

[41] Eckalbar W.L., Lasku E., Infante C.R., Elsey R.M., Markov G.J., Allen A.N., Corneveaux J.J., Logos J.B., DeNardo D.F., Huentelman M.J. (2012). Somitogenesis in anole lizard and alligator reveals evolutionary convergence and divergence in the amniote segmentation clock. Dev. Biol..

[42] Kusumi K., May C.M., Eckalbar W.L. (2013). A large-scale view of the evolution of amniote development: Insights from somitogenesis in reptiles. Curr. Opin. Genet. Dev..

[43] Nourhidayat L., Benes V., Blom S., Gomes I., Firdausi N., de Bakker M.A.G., Spaink H.P., Richardson M.K. (2025). Tokay gecko tail regeneration involves temporally collinear expression of HOXC genes and early expression of satellite cell markers. BMC Biol..

[44] Mufty S.A. (1973). Tail regeneration following amputation in adult *Triturus viridescens*. Pak. J. Zool..

[45] Iten L.E., Bryant S.V. (1976). Stages of tail regeneration in the adult newt, *Notophtalmus viridescens*. J. Exp. Zool..

[46] Ruben L.N., Johnson R.O., Clothier R.H., Balls M. (2013). Resistance to cancer in amphibians: A role for apoptosis?. Altern. Lab. Anim..

[47] Fior J. (2024). Salamander regeneration as a model for developing novel regenerative anticancer therapies. J. Cancer.

[48] Hutchins E.D., Markov G.J., Eckalbar W.L., Gorge R.M., King J.M., Tokuyama M.A., Geiger L.A., Emmert N., Ammar M.J., Allen A.P. (2014). Transcriptomic analysis of tail regeneration in the lizard *Anolis carolinensis* reveals activation of conserved vertebrate developmental and repair mechanisms. PLoS ONE.

[49] Hutchins E.D., Eckalbar W.L., Walter J.M., Mangone M., Kosumi K. (2016). Differential expression of conserved and novel microRNAs during tail regeneration in the lizard *Anolis carolinensis*. BMC Genom..

[50] Vitulo N., Dalla Valle L., Skobo T., Valle G., Alibardi L. (2017). Transcriptome analysis of the regenerating tail versus the scarring limb in lizard reveals pathways leading to successful versus unsuccessful organ regeneration in amniotes. Dev. Dyn..

[51] Vitulo N., Dalla Valle L., Skobo T., Valle G., Alibardi L. (2017). Down-regulation of lizard immuno-genes in the regenerating tail and myo-genes in the scarring limb suggests that tail regeneration occurs in an immuno-privileged organ. Protoplasma.

[52] Patel S., Ranadive I., Buch P., Khaire K., Balakrishnan S. (2022). De Novo transcriptome sequencing and analysis of differential gene expression among various stages of tail regeneration in *Hemidactylus flaviviridis*. J. Dev. Biol..

[53] Xu C., Hutchins E.D., Tokuyama M.A., Wilson-Rawls J., Kusumi K. (2020). Transcriptional analysis of scar-free wound healing during early stages of tail regeneration in the green anole lizard, *Anolis carolinensis*. J. Immunol. Reg. Med..

[54] Nagumantri S.P., Banu S., Idris M.M. (2021). Transcriptomic and proteomic analysis of *Hemidactylus frenatus* during initial stages of tail regeneration. Sci. Rep..

[55] Vonk A.C., Zhao X., Pan Z., Hudnall M.L., Oakes C.G., Lopez G.A., Hasel-Kolossa S.C., Kuncz A.W., Sengelmann S.B., Gamble D.J. (2023). Single-cell analysis of lizard blastma fibroblasts reveal phagocyte-dependent activation of hedgehog-responsive chondrogenesis. Nat. Commun..

[56] Gamble D.J., Lopez S., Yazdi M., Castro-Torres T., Lozito T.P. (2025). Probe sequencing analysis of regenerating lizard tails indicate crosstalk among osteoclasts, epidermal cells, and fibroblasts. J. Dev. Biol..

[57] Zhang J. (2012). Genetic redundancies and their evolutionary maintenance. Adv. Experim. Medic. Biol..

